# Nonequilibrium Effects on Information Recoverability of the Noisy Channels

**DOI:** 10.3390/e25121589

**Published:** 2023-11-27

**Authors:** Qian Zeng, Ran Li, Jin Wang

**Affiliations:** 1 State Key Laboratory of Electroanalytical Chemistry, Changchun Institute of Applied Chemistry, Changchun 130022, China; 2 Center for Theoretical Interdisciplinary Sciences, Wenzhou Institute, University of Chinese Academy of Sciences, Wenzhou 325001, China; 3 Department of Chemistry, State University of New York, Stony Brook, NY 11794, USA; 4 Department of Physics and Astronomy, State University of New York, Stony Brook, NY 11794, USA

**Keywords:** recoverability, entropy production, mutual information, information transfer, nonequilibrium

## Abstract

We investigated the impact of nonequilibrium conditions on the transmission and recovery of information through noisy channels. By measuring the recoverability of messages from an information source, we demonstrate that the ability to recover information is connected to the nonequilibrium behavior of the information flow, particularly in terms of sequential information transfer. We discovered that the mathematical equivalence of information recoverability and entropy production characterizes the dissipative nature of information transfer. Our findings show that both entropy production (or recoverability) and mutual information increase monotonically with the nonequilibrium strength of information dynamics. These results suggest that the nonequilibrium dissipation cost can enhance the recoverability of noise messages and improve the quality of information transfer. Finally, we propose a simple model to test our conclusions and found that the numerical results support our findings.

## 1. Introduction

The transfer of information is a crucial topic in physics, psychology, and human society, making it an important area of study for both science and technology [1,2,3,4,5]. Specifically, information theory has proven to be essential in biology, with Shannon’s pioneering work being applied to many biological systems across various scales [6,7,8]. Our central challenge is to understand how organisms can extract and represent useful information optimally from noisy channels, given physical constraints. However, the issues related to information transformation are not limited to biological systems and should be examined across a wide range of systems in physics, chemistry, and engineering. It is, therefore, essential to consider the information transfer in a more-fundamental way.

During the transmission of information, messages are frequently conveyed through noisy channels, where the noise can alter the useful information carried by the messages. Consequently, information recovery becomes a critical issue in practice [9,10,11]. Although there has been considerable research on the performance analysis of specific information recovery techniques in various fields [12,13,14,15,16], there is still a lack of a general theory on the recoverability of useful information. The quantification of recoverability should depend on the information source and the channel’s characteristics, irrespective of the specific information recovery methodologies, such as the maximum likelihood [17,18,19] or maximum a posteriori probability [20,21,22].

In [23], a classical measurement model was developed to investigate nonequilibrium behavior resulting from energy and information exchange between the environment and system. The study revealed that the Markov dynamics for sequential measurements is governed by the information driving force, which can be decomposed into two parts: the equilibrium component, which preserves time reversibility, and the nonequilibrium component, which violates time reversibility. In this work, we considered the information dynamics of noisy channels based on this nonequilibrium setup. From the perspective of nonequilibrium dynamics and thermodynamics, the information source and channel, along with their complexity, are not isolated events, but rather should be regarded as open systems [24,25]. The environment, which can exchange energy and information with these systems, can significantly influence the information transfer and its recoverability. Due to the complexity and environmental impacts, often manifesting as the stochasticity and randomness of noisy received messages, sequential information transfer should be viewed as a nonequilibrium process, leading to nonequilibrium behavior in the corresponding information dynamics. Intuitively, there must be an underlying relationship between the nonequilibrium nature and information recoverability.

This study aimed to quantify the recoverability of messages transmitted through a channel. With appropriate physical settings, we show that the randomness of the information transfer can arise from the interactions between the information receiver and the information source and from the influences of the environmental noise. This randomness can be characterized by the steady state distributions or transition probabilities of the received messages given by the receiver under different potential profiles exerted by the source, which are identified as the information driving force of the measurement dynamics [12,23]. In the sequential information transfer, the source messages randomly appear in time sequences. As a result, the receiver will change the interactions with the source messages in time. This leads to the switchings among the potential profiles and among the corresponding steady state distributions of the received messages. Then, the nonequilibrium can arise when the receiver switches its potential profiles in the sequential information transfer. We identified the nonequilibrium of the information transfer as the nonequilibrium strength, given by the difference between the information driving forces (based on the steady state transition probabilities) of the information transfer dynamics.

The main novel contributions of this work are three-fold. First, we introduce a new metric, called recoverability, to quantify the ability to recover the source messages through noisy channels. Recoverability is measured by the averaged log ratio of channel transition probabilities. Second, we demonstrate the mathematical equivalence between recoverability and entropy production [4,26,27,28,29] in nonequilibrium information dynamics. This reveals the intrinsic connection between information recoverability and thermodynamic dissipation costs. Finally, We prove that both recoverability and mutual information [30] monotonically increase with the nonequilibrium strength. This elucidates how driving a system further from equilibrium can boost information transfer quality. To our knowledge, these quantitative relationships between recoverability, entropy production, and nonequilibrium strengths have not been fully established in prior work. Our framework and analysis provide novel theoretical insights into the nonequilibrium physics of information transmission through noisy channels.

This new approach is not specific to particular models, and our conclusions are generalizable for characterizing information transfer. To further support our findings, we propose a simple information transfer model, which yields numerical results consistent with our conclusions. Lastly, we discuss the potential applications of our work in biophysics.

## 2. Information Dynamics

### 2.1. Physical Settings

Information dynamics naturally arises from information transfer, information processing, and sequential physical measurements. As discussed in the Introduction, the Markovian sequential measurement model can be properly applied to the information dynamics. Let us firstly introduce the physical settings of sequential information transfer.

**Figure 1:**
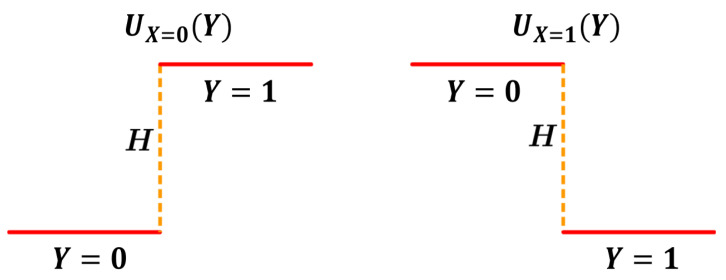
An illustration of the potential profiles $U_{X}$ in an information transfer. There are two “flat” wells in the potential: a higher well with height H>0 and a lower well with height 0. The location of the lower well depends on the source message X=0 or X=1. The left figure shows that the lower well is located at the left half of the area when X=0. The right figure shows that the lower well is located at the right half when X=1. When the receiver is found at the left half of the area, the received message is Y=0; the right half corresponds to Y=1. The locations of the lower well represent the correctly received message.

In information transfer, a receiver gains information from a source through physical interaction. The information source randomly selects a message *X* from a set X of *n* different messages, according to an a priori distribution $P_{X}$ on X. The interaction with the source causes the receiver to feel a potential *U*. The profile of *U* is determined by the message *X*, which is denoted by $U_{X}$. The potential profile $U_{X}$ contains *n* distinguishable wells with different locations, corresponding to the *n* possible messages of the source. The well with the lowest depth is unique, and its location changes as the potential profile is switched among different messages. The location of the lowest well represents the position of the current message. An illustration for the potential profile is given in Figure 1. The receiver is immersed in an environment with reciprocal temperature β.

To obtain a message $X_{i-1}$ at time i−1, the receiver interacts with the source for a period of time τ≡1. The receiver’s position in the potential *U* changes much more quickly than the source message changes. This allows the receiver to quickly reach equilibrium with the environment while the source message remains fixed during this period. The position of the receiver in the potential $U_{X_{i-1}}$ at steady state, denoted by $Y_{i}$, is viewed as the received message. If $Y_{i}$ is found at the lowest well in $U_{X_{i-1}}$, the received message is correct ($Y_{i}=X_{i-1}$). However, environmental noise can drive the receiver out of the lowest well and across the potential barriers. In this case, the receiver is found at another well located at $Y_{i}\neq X_{i-1}$, and this is a wrongly received message. The information transition probability, given by the conditional probability of *Y* when the source’s message is *X*, can be determined by the following canonical distribution:

(1) $$\begin{array}{c} q_{Y|X}\left(Y_{i}|X_{i-1}\right)=\exp\beta [F_{X_{i-1}}-U_{X_{i-1}}\left(Y_{i}\right)]. \end{array}$$

where $F_{X_{i-1}}=-\beta^{-1}\log\left\{\sum_{Y_{i}}\exp\left[-\beta U_{X_{i-1}}\left(Y_{i}\right)\right]\right\}$ denotes the free energy decided by the profile $U_{X_{i-1}}$. The information transition probability distribution $q_{Y|X}$ in Equation (1) quantifies the classical uncertainty of the information transfer channels, and it is influenced by the interaction between the receiver and the source, which is characterized by the potential $U_{X_{i-1}}$, and the environmental noise, which is characterized by the reciprocal temperature β.

In practice, the source often transmits messages randomly in time. These messages then come into a time sequence, such as $X_{1},X_{2},\ldots ,X_{t}$. The corresponding time sequence of the received messages is obtained as $Y_{2},Y_{3},\ldots ,Y_{t+1}$, where each message $Y_{i}$ is obtained from $X_{i-1}$ via the interaction described in the above. For the sake of simplicity, we assumed that the source selects message $X_{i}$ independently of the previously message $X_{i-1}$. The received message $Y_{i+1}$ is then only determined by the message $X_{i}$ according to the transition probability $q_{Y|X}\left(Y_{i+1}|X_{i}\right)$, and it is independent of both $Y_{i}$ and $X_{i-1}$. This means that the received message is not influenced by the previous received message or the previous source message. If the a priori distribution of the source messages $P_{X}$ is time-invariant, then the probability distribution of the received message is stationary, which is given by the following probability identity:

(2) $$\begin{array}{c} P_{Y}\left(Y_{i}\right)=\sum_{X_{i-1}\in X}q_{Y|X}\left(Y_{i}|X_{i-1}\right)P_{X}\left(X_{i-1}\right). \end{array}$$

### 2.2. Markovian Dynamics of Sequential Information Transfer

Since the time sequences of the source messages are assumed to be stationary and due to the ergodicity, we can formulate the sequential information transfer as a coarse-grained Markov process of the received messages within one receiving period.

When a message $Y_{i}$ has been received corresponding to the source message $X_{i-1}$, the source transmits the next message $X_{i}$, which interacts with the receiver immediately. Consequentially, the potential profile is changed from $U_{X_{i-1}}$ to $U_{X_{i}}$ promptly. Meanwhile, the receiver position in the potential remains at the previous $Y_{i}$ temporarily, and $Y_{i}$ is recognized as the initial position under the new potential $U_{X_{i}}$. The conditional joint probability of $X_{i}$ and $Y_{i}$ under the previous source message $X_{i-1}$ in this potential-switching event is given by $P\left(Y_{i},X_{i}|X_{i-1}\right)=q_{Y|X}\left(Y_{i}|X_{i-1}\right)P\left(X_{i}|X_{i-1}\right)$. Here, $P(X_{i}|X_{i-1})$ represents the transition probability from $X_{i-1}$ to $X_{i}$ and is given by $P\left(X_{i}|X_{i-1}\right)=P_{X}\left(X_{i}\right)$, because $X_{i}$ has been assumed to be independent of the previous $X_{i-1}$.

Then, the initial probability of the receiver position in the new potential profile $U_{X_{i}}$, denoted by $u^{i}\left(Y_{i}|X_{i-1},X_{i}\right)$ (it can depend on the previous source message in general cases), is given as follows: $u^{i}\left(Y_{i}|X_{i-1},X_{i}\right)=P\left(Y_{i},X_{i}|X_{i-1}\right)/P\left(X_{i}|X_{i-1}\right)=q_{Y|X}\left(Y_{i}|X_{i-1}\right)$. This means that $u^{i}$ is equal to the equilibrium distribution, which is only conditioning on the previous source message exactly.

During the message-receiving period, the source message $X_{i}$ remains unchanged within this period. The transition rates of the receiver between two possible positions *s* and $s^{\prime }$ in the potential $U_{X_{i}}$ can be represented by $r(s^{\prime }|s,X_{i})$. The Markov dynamics of the receiver position under $X_{i}$ in continuous time can be given by the following master equation $\partial_{\tau }u_{\tau }=Gu_{\tau }$, where $u_{\tau }$ is the distribution of the receiver position at time τ and *G* is the transition matrix composed of the transition rates *r*.

**Figure 2:**
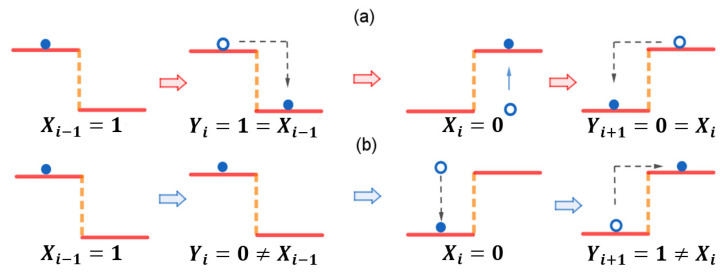
An illustration of a sequential information transfer. The time sequence of the source messages is given by $X_{i-1}=1,X_{i}=0$. The sequence of the received message is given by $Y_{i},Y_{i+1}$. (**a**) The receiver receives correct messages as $Y_{i}=1,Y_{i+1}=0$, where all the received messages appear at the correct locations of the lower wells in the potential profiles. (**b**) The receiver obtains the wrong messages as $Y_{i}=0,Y_{i+1}=1$, where all the received messages appear at the locations of the higher wells in the potential profiles.

Provided the initial distribution $u^{i}$, the solution of this master equation is given by $u_{\tau }=\exp\left(G\tau \right)u^{i}$. Since the receiver is interacting with a single environmental heat bath, then the transition rates *r* between two positions satisfy the local detailed balance condition, $r\left(s^{\prime }|s,X_{i}\right)/r\left(s|s^{\prime },X_{i}\right)=\exp\left[\beta \left(U_{X_{i}}\left(s\right)-U_{X_{i}}\left(s^{\prime }\right)\right)\right]$. The final distribution after a long enough message receiving period τ→∞ can achieve the equilibrium distribution, i.e., $u^{f}=\lim_{\tau \to \infty }\exp\left(G\tau \right)u^{i}=q_{Y|X=X_{i}}$. Here, we take τ=1 for the long enough time to reach the equilibrium. At the end of the period, we take the position $Y_{i+1}$ as the new received message. An illustration of the sequential information transfer is given in Figure 2.

According to the continuous-time dynamics, the matrix $K=\lim_{\tau \to \infty }\exp\left(G\tau \right)$ is recognized as the transition probability matrix for the information transfer dynamics at a coarse-grained level. The transition probabilities can be given by $K\left(Y_{i+1},Y_{i}\right)\equiv k\left(Y_{i+1}|Y_{i},X_{i}\right)=qY|X\left(Y_{i+1}|X_{i}\right)$, which is recognized as the information driving force [4,29] from the initial position $Y_{i}$ to the final one $Y_{i+1}$ within a period.

Due to the description in the above, we obtain the master equation of this coarse-grained Markov process in discrete time as follows:

(3) $$\begin{array}{c} u^{f}\left(Y_{i+1}|X_{i}\right)=\sum_{Y_{i}}k\left(Y_{i+1}|Y_{i},X_{i}\right)u^{i}\left(Y_{i}|X_{i}\right), \end{array}$$

where

$$\begin{array}{c} \left\{\begin{array}{cc} u^{f}\left(Y_{i+1}|X_{i}\right)=q_{Y|X}\left(Y_{i+1}|X_{i}\right), & \mathrm{final}\;\mathrm{equilibrium}\;\mathrm{distribution},\\ k\left(Y_{i+1}|Y_{i},X_{i}\right)=q_{Y|X}\left(Y_{i+1}|X_{i}\right), & \mathrm{information}\;\mathrm{driving}\;\mathrm{force},\\ u^{i}\left(Y_{i}|X_{i}\right)=q_{Y|X}\left(Y_{i}|X_{i-1}\right), & \mathrm{initial}\;\mathrm{distribution}. \end{array}\right. \end{array}$$

## 3. Information Recoverability

### 3.1. Decision Rules of Information Recovery

In this section, we will discuss two decision rules used to recover information. Due to the stochasticity of the noisy channel, two different source messages $X_{i-1}$ and $X_{i-1}^{\prime }$ can be both transformed into the same received message $Y_{i}$ by the channel, according to the information driving forces $q_{Y|X}\left(Y_{i}|X_{i-1}\right)$ and $q_{Y|X}\left(Y_{i}|X_{i-1}^{\prime }\right)$ (see Equations (1) and (3)), respectively. If one extracts the original message $X_{i-1}$ from the received message $Y_{i}$, a decision rule should be employed to justify that $Y_{i}$ originated from $X_{i-1}$ rather than another message $X_{i-1}^{\prime }\neq X_{i-1}$.

There are mainly two kinds of decision rules frequently used for recovering the source messages. The first kind is the maximum likelihood rule, that is choosing the message $X_{i-1}$ such that

(4) $$\begin{array}{c} l_{Y_{i}}\left(X_{i-1},X_{i-1}^{\prime }\right)=\log\frac{q_{Y|X}\left(Y_{i}|X_{i-1}\right)}{q_{Y|X}\left(Y_{i}|X_{i-1}^{\prime }\right)}>0,\;\mathrm{for}\;\mathrm{all}\;X_{i-1}^{\prime }\neq X_{i-1}. \end{array}$$

The maximum likelihood rule is suitable for the cases where the a priori distribution $P_{X}$ is uniform or $P_{X}$ is not important for the information transfer.

The second kind of decision rule is the so-called maximum a posteriori probability. This means selecting the message $X_{i-1}$ such that

(5) $$\begin{array}{c} r_{Y_{i}}\left(X_{i-1},X_{i-1}^{\prime }\right)=\log\frac{P_{X|Y}\left(X_{i-1}|Y_{i}\right)}{P_{X|Y}\left(X_{i-1}^{\prime }|Y_{i}\right)}>0,\;\mathrm{for}\;\mathrm{all}\;X_{i-1}^{\prime }\neq X_{i-1}. \end{array}$$

where the a posteriori probability follows the Bayes equation as

(6) $$\begin{array}{c} P_{X|Y}\left(X_{i-1}|Y_{i}\right)=\frac{P(X_{i-1},Y_{i})}{P_{Y}\left(Y_{i}\right)}=\frac{q_{Y|X}\left(Y_{i}|X_{i-1}\right)P_{X}\left(X_{i-1}\right)}{\sum_{X_{i-1}}q_{Y|X}\left(Y_{i}|X_{i-1}\right)P_{X}\left(X_{i-1}\right)}. \end{array}$$

If the a priori distribution $P_{X}$ is not uniform and $P_{X}$ carries the significant information of the message, then the maximum a posteriori probability rule is more appropriate than the maximum likelihood rule for the message recovery.

The connection of these two decision rules is that the log ratio *r* in Equation (5) carries additional information about the a priori distribution $P_{X}$ compared to the log ratio *l* in Equation (4) as follows:

(7) $$\begin{array}{c} r_{Y_{i}}\left(X_{i-1},X_{i-1}^{\prime }\right)=l_{Y_{i}}\left(X_{i-1},X_{i-1}^{\prime }\right)+\gamma , \end{array}$$

where $\gamma =\log\frac{P_{X}\left(X_{i-1}\right)}{P_{X}\left(X_{i-1}^{\prime }\right)}$ is the log ratio of the a priori probabilities. When the message $X_{i-1}$ is transmitted and the log ratio *l* satisfies Equation (4) or *r* satisfies Equation (5), then $X_{i-1}$ can be recovered correctly. Otherwise, if another message ${\hat{X}}_{i-1}\neq X_{i-1}$ maximizes the likelihood or the a posteriori probability, then ${\hat{X}}_{i-1}$ rather than the original message $X_{i-1}$ can be chosen by the decision rules incorrectly.

### 3.2. Information Recoverability of Noisy Channels

The decision rules can be used to justify the transferred information. However, the ability of the noisy channels to recover the transferred information is still unclear. We need a new physical quantity to quantify the recoverability of the source messages. Obviously, this quantity should be independent of the decision rules. In this subsection, we will discuss the definition of information recoverability for the noisy channels.

For illustration, we firstly considered the maximum likelihood decision rule. One should be aware that the information driving force or transition probability $q_{Y|X}$ in Equation (1) works as the key characterization to decide the performances of the decision rules, if the a priori distribution $P_{X}$ is fixed. From the perspective of information theory, the log ratio *l* in Equation (4) is a fundamental entity to quantify the recoverability of the source messages, because *l* merely depends on the information driving force $q_{Y|X}$. Intuitively, while the a priori distribution $P_{X}$ is fixed, the inequality $l_{Y_{i}}\left(X_{i-1},X_{i-1}^{\prime }\right)\leq 0$ indicates the situation that $X_{i-1}$ is completely unrecoverable. On the other hand, as $l_{Y_{i}}\left(X_{i-1},X_{i-1}^{\prime }\right)$ increases in the positive regime, the a posteriori distribution $P_{X|Y}$ (see Equation (6)) conditioned on $Y_{i}$ tends to be more concentrated on $X_{i-1}$ than $X_{i-1}^{\prime }\neq X_{i-1}$, and hence, $X_{i-1}$ becomes more recoverable. On the contrary, as $l_{Y_{i}}\left(X_{i-1},X_{i-1}^{\prime }\right)$ decreases, then $X_{i-1}$ becomes less recoverable when $Y_{i}$ is received.

To address our idea more clearly, we rewrite $l_{Y_{i}}\left(X_{i-1},X_{i-1}^{\prime }\right)$ in the following form:

(8) $$\begin{array}{c} l_{Y_{i}}\left(X_{i-1},X_{i-1}^{\prime }\right)=i\left(X_{i-1},Y_{i}\right)+i_{e}\left(X_{i-1}^{\prime },Y_{i}\right). \end{array}$$

with

(9) $$\begin{array}{c} \left\{\begin{array}{cc} i\left(X_{i-1},Y_{i}\right)=\log\frac{q_{Y|X}\left(Y_{i}|X_{i-1}\right)}{P_{Y}\left(Y_{i}\right)}, & \mathrm{stochastic}\;\mathrm{mutual}\;\mathrm{information},\;\\ i_{e}\left(X_{i-1}^{\prime },Y_{i}\right)=-\log\frac{q_{Y|X}\left(Y_{i}|X_{i-1}^{\prime }\right)}{P_{Y}\left(Y_{i}\right)}, & \mathrm{stochastic}\;\mathrm{error}\;\mathrm{information},\\ P_{Y}\left(Y_{i}\right)\mathrm{is}\;\mathrm{given}\;\mathrm{in}\;\mathrm{Equation}\;(2). \end{array}\right. \end{array}$$

Here, $i(X_{i-1},Y_{i})$ and $i_{e}\left(X_{i-1}^{\prime },Y_{i}\right)$ quantify the stochastic mutual information of the messages $X_{i-1}$ and $X_{i-1}^{\prime }$ contained in the received message $Y_{i}$, respectively. When $X_{i-1}$ is transmitted and $Y_{i}$ is received, $i(X_{i-1},Y_{i})$ is the useful information for recovering $X_{i-1}$. On the other hand, $i_{e}\left(X_{i-1}^{\prime },Y_{i}\right)$ quantifies the noise-induced error, which introduces a spurious correlation between $Y_{i}$ and $X_{i-1}^{\prime }$. The negative sign in $i_{e}$ represents the part of the useful information from the correct source message $X_{i-1}$ that is reduced by the error. Then, the log ratio *l* quantifies the remaining useful information of $X_{i-1}$ while the error information $i(X_{i-1}^{\prime },Y_{i})$ reduces the useful information $i(X_{i-1},Y_{i})$. The larger *l* becomes, the more useful information of the source message $X_{i-1}$ is preserved in the received message $Y_{i}$, and $X_{i-1}$ becomes more recoverable.

With this consideration, we used the average of the log ratio *l* to describe the overall recoverability of the source messages, i.e., we can properly define information recoverability *R* for the maximum likelihood decision rule as follows:

(10) $$\begin{array}{ccc} R & = & \langle l_{Y_{i}}\left(X_{i-1},X_{i-1}^{\prime }\right)\rangle \\ & = & \sum_{X_{i-1},X_{i-1}^{\prime },Y_{i}}P_{X}\left(X_{i-1}\right)P_{X}\left(X_{i-1}^{\prime }\right)q_{Y|X}\left(Y_{i}|X_{i-1}\right)\log\frac{q_{Y|X}\left(Y_{i}|X_{i-1}\right)}{q_{Y|X}\left(Y_{i}|X_{i-1}^{\prime }\right)}, \end{array}$$

where the average is taken over the ensembles of $X_{i-1}$, $X_{i-1}^{\prime }$, and $Y_{i}$.

Now, let us discuss the information recoverability for the maximum a posteriori probability decision rule. It can be verified that the average of the log ratio of the a posteriori probabilities *r* in the maximum a posteriori probability rule (see Equation (5)) is equal to the recoverability *R*, i.e., $R=\langle r_{Y_{i}}\left(X_{i-1},X_{i-1}^{\prime }\right)\rangle$, with the log ratio of the a priori probabilities γ (see Equation (7)) vanishing in the average. This implies that the entity *R* can be used as a new rationale for the characterization of the recoverability, which should not depend on concrete decision rules.

### 3.3. Information Transfer Rate Enhanced by Recoverability

In this subsection, we derive the novel result that the information transfer rate increases monotonically with recoverability, formally proving that enhancing the ability to recover messages also boosts the rate of reliable information transmission through noisy channels.

The relationship between the information transfer rate and the recoverability can be given by Equations (8) and (9) straightforwardly:

(11) $$\begin{array}{ccc} R & = & \langle l_{Y_{i}}\left(X_{i-1},X_{i-1}^{\prime }\right)\rangle \\ & = & \langle i\left(X_{i-1},Y_{i}\right)+i_{e}\left(X_{i-1}^{\prime },Y_{i}\right)\rangle \\ & = & I+I_{e}\geq 0 \end{array}$$

with

(12) $$\begin{array}{c} \left\{\begin{array}{c} I=\langle i\left(X_{i-1},Y_{i}\right)\rangle =\sum_{X_{i-1},Y_{i}}P_{X}\left(X_{i-1}\right)q_{Y|X}\left(Y_{i}|X_{i-1}\right)\log\frac{q_{Y|X}\left(Y_{i}|X_{i-1}\right)}{P_{Y}\left(Y_{i}\right)}\geq 0\\ I_{e}=\langle i_{e}\left(X_{i-1}^{\prime },Y_{i}\right)\rangle =\sum_{X_{i-1}^{\prime },Y_{i}}P_{X}\left(X_{i-1}^{\prime }\right)P_{Y}\left(Y_{i}\right)\log\frac{q_{Y|X}\left(Y_{i}|X_{i-1}^{\prime }\right)}{P_{Y}\left(Y_{i}\right)}\geq 0 \end{array}\right. \end{array}$$

Here, *I* is recognized as the mutual information between the time sequences of the messages $X=\{X_{1},X_{2},\ldots ,X_{t}\}$ and $Y=\{Y_{1},Y_{2},\ldots ,Y_{t}\}$; $I_{e}$ is the averaged error information of the information dynamics with the meaning of $I_{e}$ interpreted in Equations (8) and (9). Since *I* and $I_{e}$ can be given in terms of the relative entropies as $I=D_{KL}\{P\left(X_{i-1},Y_{i}\right)\left|\right|P_{Y}\left(Y_{i}\right)P_{X}\left(X_{i-1}\right)\}$ and $I_{e}=D_{KL}\{P\left(X_{i-1}^{\prime },Y_{i}\right)\left|\right|P_{Y}\left(Y_{i}\right)P_{X}\left(X_{i-1}^{\prime }\right)\}$, then *I* and $I_{e}$ are both positively defined. Thus, the recoverability *R* is a nonnegative valued, due to Equation (11).

The mutual information *I* quantifies the useful information of the source messages contained in the received messages and characterizes the rate of the information transfer. It is also called the information transfer rate. Equation (11) implies that the information transfer rate *I* can be given as a function of the recoverability:

(13) $$\begin{array}{c} I=R-I_{e}. \end{array}$$

We can verify that *I* is a monotonically increasing function with respect to *R* with a fixed a priori distribution $P_{X}$. This is because both the mutual information *I* and recoverability *R* are a convex function of the information driving force $q_{Y|X}$ when $P_{X}$ is fixed (see [31] and Appendix A of this paper). They achieve the same minimum value of 0 at the points $q_{Y|X}\left(Y_{i}|X_{i-1}\right)=q_{Y|X}\left(Y_{i}|X_{i-1}^{\prime }\right)$, where all the source messages cannot be distinguished from each other. Then, *I* and *R* are both monotonically increasing functions in the same directions as $q_{Y|X}$. Therefore, *I* is a monotonically increasing function of *R*.

From the convexity of the recoverability, one can easily obtain the following two observations. The first observation is that, if the source and received messages $X_{i-1}$ and $Y_{i}$ are independent of each other, then all the source messages become unrecoverable. Otherwise, *R* increases monotonically while each absolute difference between two information driving forces $|d_{Y_{i}}\left(X_{i-1},X_{i-1}^{\prime }\right)|$ increases (see Appendix A). Here, the difference *d* is defined as

(14) $$\begin{array}{c} d_{Y_{i}}\left(X_{i-1},X_{i-1}^{\prime }\right)=\frac{1}{2}\left[q_{Y|X}\left(Y_{i}|X_{i-1}\right)-q_{Y|X}\left(Y_{i}|X_{i-1}^{\prime }\right)\right]. \end{array}$$

On the other hand, the information transfer rate *I* is also a convex function over all the possible information driving forces $q_{Y|X}$ [32]. The proof of the convexity of *I* can be given by applying the log sum inequality. The unique minimum of *I* is provided by zero, and this minimum information transfer rate can be achieved if and only if the messages $X_{i-1}$ and $Y_{i}$ are independent of each other, i.e., $d_{Y_{i}}\left(X_{i-1},X_{i-1}^{\prime }\right)=0$ for all $X_{i-1}^{\prime }\neq X_{i-1}$. Thus, both the information transfer rate *I* and the recoverability *R* increase monotonically as each absolute difference $|d_{Y_{i}}\left(X_{i-1},X_{i-1}^{\prime }\right)|$ increases. This indicates that the information transfer can be enhanced by the recoverability monotonically due to Equation (13). The increase in the information transfer rate also implies the improvement of the information recovery, with smaller upper bounds of the error probabilities of the decision rules in Equations (4) and (5) [33].

## 4. Nonequilibrium Information Dynamics

In this section, we will develop the model of nonequilibrium information dynamics by introducing the time-reverse sequence of the sequential information transfer process. We will show that the recoverability is closely related to the nonequilibrium behavior of the Markovian information dynamics described in Equation (3). As we can see from the following discussions, the difference *d* given in Equation (14) works as the nonequilibrium information driving force of the information dynamics. The recoverability *R* can be shown as the thermodynamic dissipation cost or the entropy production of the information dynamics, driven by the nonequilibrium information driving force *d*.

The nonequilibrium behavior of the Markovian information dynamics (Equation (3)) can be seen intuitively from the following fact: When the source message is changed from $X_{i-1}$ to $X_{i}$, the profile of the potential is changed from $U_{X_{i-1}}$ to $U_{X_{i}}$ accordingly. Since $U_{X_{i}}$ is different from $U_{X_{i-1}}$ if $X_{i}\neq X_{i-1}$, the corresponding equilibrium distribution of the receiver message $q_{Y|X=X_{i}}$, which is conditioning on $X_{i}$, can differ from the previous equilibrium distribution $q_{Y|X=X_{i-1}}$, which is conditioning on $X_{i-1}$. This indicates that the receiver is driven out of equilibrium in the new potential $U_{X_{i}}$ at the beginning of the message-receiving period. Then, the difference between two information driving forces under different potential profiles at the same received message, where the potential profile is switched, can reflect the degree of this nonequilibrium. With this consideration, we decompose the information driving force in Equation (3) into two parts [4,29]:

(15) $$\begin{array}{c} q_{Y|X}\left(Y_{i}|X_{i-1}\right)=d_{Y_{i}}\left(X_{i-1},X_{i}\right)+m_{Y_{i}}\left(X_{i-1},X_{i}\right), \end{array}$$

with

(16) $$\begin{array}{c} \left\{\begin{array}{cc} d_{Y_{i}}\left(X_{i-1},X_{i}\right)=\frac{1}{2}\left[q_{Y|X}\left(Y_{i}|X_{i-1}\right)-q_{Y|X}\left(Y_{i}|X_{i}\right)\right], & \mathrm{nonequilibrium}\;\mathrm{strength},\\ m_{Y_{i}}\left(X_{i-1},X_{i}\right)=\frac{1}{2}\left[q_{Y|X}\left(Y_{i}|X_{i-1}\right)+q_{Y|X}\left(Y_{i}|X_{i}\right)\right], & \mathrm{equilibrium}\;\mathrm{strength}. \end{array}\right. \end{array}$$

In Equation (15), if the nonequilibrium strength *d* vanishes at every source and received message, then the information transfer process (Equation (3)) is at the equilibrium state, and the information driving force $q_{Y|X}$ degenerates to its equilibrium strength *m*. In this situation, the new potential $U_{X_{i}}$ is equal to the previous potential $U_{X_{i-1}}$ plus a constant *a*, i.e., $U_{X_{i}}=U_{X_{i-1}}+a$, and the receiver stays at the same equilibrium state under $X_{i}$ as that under $X_{i-1}$. Then, the nonequilibriumness in the information transfer vanishes, and the vanishing nonequilibrium strength $d_{0}=0$ at every source and received message is called the equilibrium point of the information transfer. Consequentially, the equilibrium point makes any two different source message $X_{i}$ and $X_{i-1}$ indistinguishable from each other by the receiver (see the *Decision Rules of Information Recovery* Section). Clearly, this leads to invalid information transfer. On the other hand, the non-vanishing nonequilibrium strength d≠0 indicates that not only the information transfer is a nonequilibrium process, but also some source message *X* can be distinguished from the others by the receiver.

In addition, the nonequilibrium strength *d* shows the same form as Equation (14), where we can regard the source message $X_{i-1}^{\prime }$ in Equation (14) as $X_{i}$ in Equation (16) because the source select the message in the i.i.d. way. This *d* guarantees the convexity of the information recoverability (Equation (10), Appendix A of this paper). This means that the information recoverability is a non-decreasing function of the absolute nonequilibrium strength. Then, as a consequence, the information system is further driven away from the equilibrium state, which will give rise to the better recoverability. This leads to the connection between the nonequilibrium thermodynamics and the recoverability.

## 5. Nonequilibrium Information Thermodynamics

The nonequilibrium behavior of a system can give rise to a thermodynamic dissipation cost in the form of energy, matter, or information, which can be quantified by the entropy production [31,34,35]. In this section, we will discuss the entropy production of the nonequilibrium information dynamics. It is shown that the entropy production can be used to characterize the averaged dissipative information during the nonequilibrium process.

The entropy production can be written in terms of the work performed on the receiver. From the perspective of stochastic thermodynamics [18], for receiving an incoming message $X_{i}$, a stochastic work $w=U_{Xi}\left(Y\left(i\right)\right)-U_{Xi-1}\left(Y\left(i\right)\right)$ should be performed on the receiver to change the potential profile from $U_{Xi-1}$ to $U_{Xi}$. Meanwhile, the free energy change $\Delta F=F_{Xi-1}-F_{Xi}$ (the free energy is shown in Equation (1) quantifies the work to change the potential profile within an equilibrium information transfer. The difference between *w* and ΔF quantifies the energy dissipation within a nonequilibrium information transfer, which is recognized as the stochastic entropy production when receiving one single message. This stochastic entropy production can be shown as the logarithmic ratio between two transition probabilities at the same received message, as follows:

(17) $$\begin{array}{c} \sigma =\beta \left(w-\Delta F\right)=\log\frac{q_{Y|X}\left(Y_{i}|X_{i-1}\right)}{q_{Y|X}\left(Y_{i}|X_{i}\right)}, \end{array}$$

where

$$\begin{array}{c} \left\{\begin{array}{cc} w=U_{Xi}\left(Y\left(i\right)\right)-U_{Xi-1}\left(Y\left(i\right)\right), & \mathrm{stochastic}\;\mathrm{work},\\ \Delta F=F_{Xi-1}-F_{Xi}, & \mathrm{free}\;\mathrm{energy}\;\mathrm{difference}. \end{array}\right. \end{array}$$

On the other hand, the receiver can be regarded as a finite-state information storage. It stores the information from the source message, which may be corrupted by environmental noise. However, due to its finite memory, the receiver must erase the stored information of the source message $X_{i-1}$ when a new source message $X_{i}$ is coming. According to the theory of information thermodynamics [1,2,3,4,5], the work *w* is needed to erase the information of $X_{i-1}$ and to write the new information of $X_{i}$ in the receiver.

The information of the source message $X_{i-1}$ stored in the receiver is quantified by the stochastic mutual information between the previous received message $Y_{i}$ and $X_{i-1}$, $i=\log\frac{q_{Y|X}\left(Y_{i}|X_{i-1}\right)}{P_{Y}\left(Y_{i}\right)}$ (see Equation (9)). On the other hand, not all the work is used to erase the information. Due to environmental noise, a part of the work introduces error information, which shows the spurious correlation between $Y_{i}$ and the incoming source message $X_{i}$, quantified by the negative stochastic mutual information $i_{e}=-\log\frac{q_{Y|X}\left(Y_{i}|X_{i}\right)}{P_{Y}\left(Y_{i}\right)}$ (see Equation (9)). Then, it can be shown that the stochastic entropy production in Equation (17) can be rewritten as the sum of the useful information *i* and the error information $i_{e}$:

(18) $$\begin{array}{c} \sigma =i+i_{e}, \end{array}$$

where

$$\begin{array}{c} \left\{\begin{array}{cc} i=\log\frac{q_{Y|X}\left(Y_{i}|X_{i-1}\right)}{P_{Y}\left(Y_{i}\right)}, & \mathrm{stochastic}\;\mathrm{mutual}\;\mathrm{information},\;\\ i_{e}=-\log\frac{q_{Y|X}\left(Y_{i}|X_{i}\right)}{P_{Y}\left(Y_{i}\right)}, & \mathrm{stochastic}\;\mathrm{error}\;\mathrm{information},\\ P_{Y}\left(Y_{i}\right)\mathrm{is}\;\mathrm{given}\;\mathrm{in}\;\mathrm{Equation}\;(2). \end{array}\right. \end{array}$$

Equation (18) indicates that the stochastic entropy production σ not only quantifies the work dissipation in one single information transfer, but also justifies the detailed recoverability of each received messages, as shown in Equation (8).

The average of the stochastic entropy production Σ=〈σ〉 is the key characterization of a nonequilibrium process in thermodynamics, which is given as follows:

(19) $$\begin{array}{ccc} \Sigma & = & \beta (W-\Delta F)\\ & = & I+I_{e}\\ & = & \sum_{Y_{i},X_{i-1},X_{i}}P_{X}\left(X_{i-1}\right)P_{X}\left(X_{i}\right)q_{Y|X}\left(Y_{i}\right|X_{i-1}\log\frac{q_{Y|X}\left(Y_{i}|X_{i-1}\right)}{q_{Y|X}\left(Y_{i}|X_{i}\right)}\geq 0, \end{array}$$

where

$$\begin{array}{c} \left\{\begin{array}{cc} W=\langle w\rangle =\beta^{-1}\Sigma , & \mathrm{averaged}\;\mathrm{work},\;\\ \Delta F=\langle \Delta F\rangle , & \mathrm{averaged}\;\mathrm{free}\;\mathrm{energy}\;\mathrm{difference}\;\mathrm{equals}\;0\;\mathrm{in}\;\mathrm{this}\;\mathrm{case},\\ I=\langle i\rangle =\sum_{X_{i-1},Y_{i}}P_{X}\left(X_{i-1}\right)q_{Y|X}\left(Y_{i}|X_{i-1}\right)\log\frac{q_{Y|X}\left(Y_{i}|X_{i-1}\right)}{P_{Y}\left(Y_{i}\right)}\geq 0, & \mathrm{useful}\;\mathrm{information}\;\mathrm{of}\;X_{i-1},\\ I_{e}=\langle i_{e}\rangle =\sum_{X_{i},Y_{i}}P_{X}\left(X_{i}\right)P_{Y}\left(Y_{i}\right)\log\frac{P_{Y}\left(Y_{i}\right)}{q_{Y|X}\left(Y_{i}|X_{i}\right)}\geq 0, & \mathrm{error}\;\mathrm{information}\;\mathrm{from}\;X_{i}. \end{array}\right. \end{array}$$

Equation (19) expresses the first equality in terms of the averaged energy or work dissipation (see Equation (17)). Since the averaged free energy ΔF vanishes in this case, then the averaged work *W* is completely converted into heat and dissipated into the environment. Since the averaged entropy production Σ is shown as the relative entropy $D_{KL}(q_{Y|X=X_{i-1}}\left|\right|q_{Y|X=X_{i}})$ (the third equality in Equation (19)), then Σ is always nonnegative, which is equivalently shown as the work bound W≥ΔF=0. This is the thermodynamic second law for the information transfer. Here, the equal sign in the second law implies that the information transfer process is at the equilibrium point d=0 (see Equation (16)) and the discussion below). Otherwise, if Σ>0, then the measurement is at a nonequilibrium point.

On the other hand, the second equality in Equation (19) establishes the bridge between the nonequilibrium energy dissipation and the information transfer. The entropy production is taken as the sum of the useful information, quantified by the mutual information *I* between the source and received information, and the error information $I_{e}$. This expression shows that the entropy production works as the recoverability given in Equation (10):

(20) $$\begin{array}{c} \Sigma =R. \end{array}$$

This equation shows a novel result that recoverability is equivalent to entropy production. This means that information retrieval is linked to thermodynamic costs. In other words, improving the recoverability means increasing the nonequilibrium dissipation cost.

In addition, since both *I* and $I_{e}$ are shown as the relative entropies in the forms of $D_{KL}(q_{Y|X}\left|\right|P_{Y})\geq 0$ and $D_{KL}(P_{Y}\left|\right|q_{Y|X})\geq 0$, then *I* and $I_{e}$ are both nonnegative. It is seen that the error information $I_{e}\geq 0$ works as the interference, which reduces the recoverability of information transfer and enlarges the energy dissipation. For this reason, the work bound in the second law can be given more tightly by the acquired information $\beta^{-1}I$, shown as

(21) $$\begin{array}{c} W\geq \beta^{-1}I\geq 0. \end{array}$$

This lower bound indicates that, for valid information transfer I>0, we should perform a positive work of $\beta^{-1}I$ at least to erase the previous transmitted information stored in the receiver. Here, Equation (21) is the so-called generalized Landauer principle [3,4], which gives the minimum work or the minimum entropy production estimation for valid information erasing and transfer in this model on the average level. Since both the entropy production and mutual information are a monotonically increasing function of the nonequilibrium strength, the entropy production or the work performed on the receiver is a monotonically increasing function of the valid information transfer. This indicates that the more-useful information of one source message is transferred, and more energy or work dissipation is needed.

## 6. Numerical Results

In this section, we will present a simple example that can test our previous conclusions numerically.

At first, we set the source messages to be $X_{i}=1,2,3$ and the possible received messages $Y_{i}=1,2,3$. The a priori distribution $P_{X}$ is given by the following column vector:

(22) $$\begin{array}{c} P_{X}={\left[P_{X}\left(1\right);P_{X}\left(2\right);P_{X}\left(3\right)\right]}^{T}={\left[0.4610;0.0753;0.4637\right]}^{T}. \end{array}$$

The information driving force or the transition probabilities are given by the following matrix:

(23) $$\begin{array}{c} q_{Y|X}=\left[\begin{array}{ccc} 0.4410 & 0.5558 & 0.1562\\ 0.4903 & 0.3848 & 0.3067\\ 0.0687 & 0.0594 & 0.5371 \end{array}\right], \end{array}$$

where $q_{Y|X}$ follows a given potential, which is shown in Equation (1)), and the labels of the columns and rows of $q_{Y|X}$ represent the indices of the source and received messages, respectively. We then evaluated the recoverability *R* along a stochastic time sequence of the source and received messages, $Z=\{\left(X_{1},Y_{2}\right),\left(X_{2},Y_{3}\right),\ldots \left(X_{t},Y_{t+1}\right)\}$. This time sequence *Z* is generated by the given probabilities in Equations (22,23). Following the Markov nature, the probabilities of *Z* can be given by $P\left(Z\right)=\prod_{i=1}^{t}P_{X}\left(X_{i}\right)q_{Y|X}\left(Y_{i+1}|X_{i}\right)$. If the time *t* is long enough, then the sequence *Z* will enter the typical set of the joint sequences of the source and received messages [31]. This means that all the time averages of *Z* will converge to the typical statistics of the joint sequences of the source and received messages. However, if we generate another time sequence $Z^{\prime }=\left\{\left(X_{1}^{\prime },Y_{2}\right),\left(X_{2}^{\prime },Y_{3}\right),\ldots \left(X_{t}^{\prime },Y_{t+1}\right)\right\}$, where the sequence of the received messages is the same as that in the typical sequence *Z*, but with the original sequence of the source messages being randomly shuffled, we then obtain a non-typical sequence $Z^{\prime }$ with probability $P\left(Z^{\prime }\right)=\prod_{i=1}^{t}P_{X}\left(X_{i}^{\prime }\right)q_{Y|X}\left(Y_{i+1}|X_{i}^{\prime }\right)$. The recoverability *R* in the information transfer can be evaluated by the time average of the log ratio of the probabilities P(Z) to $P(Z^{\prime })$:

$$\begin{array}{c} R^{\prime }=\frac{1}{t}\log\frac{P(Z)}{P(Z^{\prime })},\;\mathrm{for}\;\mathrm{long}\;\mathrm{enough}\;t. \end{array}$$

On the other hand, we can evaluate the average of the stochastic entropy production in Equation (17) along the same time sequence *Z* as follows:

$$\begin{array}{c} \Sigma^{\prime }=\frac{1}{t}\sum_{i=2}^{t}\log\frac{q_{Y|X}\left(Y_{i}|X_{i-1}\right)}{q_{Y|X}\left(Y_{i}|X_{i}\right)},\;\mathrm{for}\;\mathrm{long}\;\mathrm{enough}\;t. \end{array}$$

**Figure 3:**
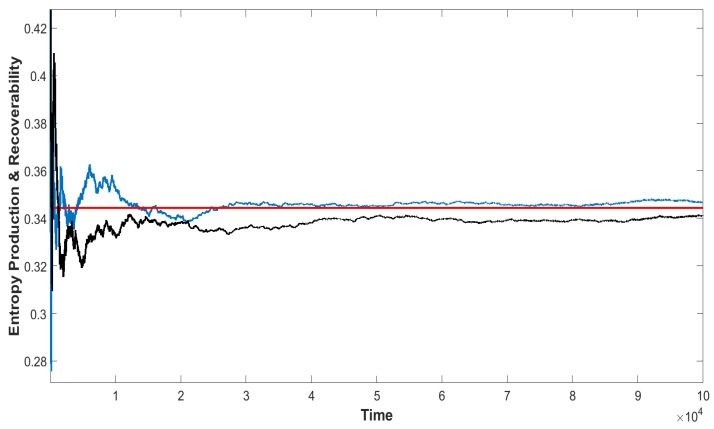
Equivalence of the entropy production and the recoverability. Black line, the time average of the stochastic entropy production. Blue line, the time average of the stochastic recoverability. Red line, the theoretical value. Both the entropy production and the recoverability converge to the theoretical value with time.

The entropy production $\Sigma^{\prime }$ converges to the same theoretical value with the recoverability $R^{\prime }$ (Equation (10)) in the long time limit. The related results are shown Figure 3, which verify the fact that the thermodynamic dissipation cost works as the recoverability.

We demonstrate that both the mutual information *I* (Equation (12)) and the recoverability or the entropy production (Equation (19)) are convex functions of the nonequilibrium strength *d* (Equations (14) and (16), Appendix A of this paper). Due to the i.i.d. assumption in the *Physical Settings* Section, we can drop the time indices in the information driving force $q_{X|Y}$. Then, the nonequilibrium decomposition of $q_{X|Y}$ in Equation (15) becomes

(24) $$\begin{array}{c} q_{Y|X}\left(y|x\right)=m_{y}\left(x,x^{\prime }\right)+d_{y}\left(x,x^{\prime }\right),\;\mathrm{for}\;x^{\prime }\neq x \end{array}$$

with

(25) $$\begin{array}{c} \left\{\begin{array}{c} m_{y}\left(x,x^{\prime }\right)=\frac{1}{2}\left[q_{Y|X}\left(y|x\right)+q_{Y|X}\left(y|x^{\prime }\right)\right],\\ d_{y}\left(x,x^{\prime }\right)=\frac{1}{2}\left[q_{Y|X}\left(y|x\right)-q_{Y|X}\left(y|x^{\prime }\right)\right]. \end{array}\right. \end{array}$$

where *m* and *d* are recognized as the equilibrium and nonequilibrium strengths, respectively.

By substituting Equation (24) into the expressions of *I* (Equation (12)) and *R* (Equation (19)), respectively, we have that

(26) $$\begin{array}{c} \left\{\begin{array}{c} I=\sum_{x,y}P_{X}\left(x\right)\left[m_{y}\left(x,x^{\prime }\right)+d_{y}\left(x,x^{\prime }\right)\right]\log\frac{m_{y}\left(x,x^{\prime }\right)+d_{y}\left(x,x^{\prime }\right)}{\sum_{x}P_{X}\left(x\right)\left[m_{y}\left(x,x^{\prime }\right)+d_{y}\left(x,x^{\prime }\right)\right]}\\ R=\sum_{x,x^{\prime },y}P_{X}\left(x^{\prime }\right)P_{X}\left(x\right)d_{y}\left(x,x^{\prime }\right)\log\frac{m_{y}\left(x,x^{\prime }\right)+d_{y}\left(x,x^{\prime }\right)}{m_{y}\left(x,x^{\prime }\right)-d_{y}\left(x,x^{\prime }\right)} \end{array}\right. \end{array}$$

Here, we note that the total number of source messages need not be equal to that of the received messages in practice. Under this consideration, we set the source messages to be x=1,2 (2 messages) and the possible received messages to be y=1,2,3 (3 messages). Then, the information driving force $q_{Y|X}$ becomes a 3×2 matrix, which can be given by

$$\begin{array}{c} q_{Y|X}=\left[\begin{array}{cc} q_{Y|X}\left(1|1\right) & q_{Y|X}\left(1|2\right)\\ q_{Y|X}\left(2|1\right) & q_{Y|X}\left(2|2\right)\\ q_{Y|X}\left(3|1\right) & q_{Y|X}\left(3|2\right) \end{array}\right]. \end{array}$$

According to the nonequilibrium decomposition given in Equation (24), for this case, we have the equilibrium and nonequilibrium strengths as follows:

$$\begin{array}{c} \left\{\begin{array}{c} m_{y}=m_{y}\left(1,2\right)=m_{y}\left(2,1\right)=\frac{1}{2}\left[q_{Y|X}\left(y|1\right)+q_{Y|X}\left(y|2\right)\right],\\ d_{y}=d_{y}\left(1,2\right)=-d_{y}\left(2,1\right)=\frac{1}{2}\left[q_{Y|X}\left(y|1\right)-q_{Y|X}\left(y|2\right)\right]. \end{array}\right. \end{array}$$

Due to the nonnegativity and the normalization of the conditional probabilities, i.e., $q_{Y|X}\geq 0$ and $\sum_{y}q_{Y|X}\left(y|x\right)=1$, the constraints on *m* and *d* can be given as follows:

(27) $$\begin{array}{c} m_{y}\geq 0,\;\sum_{y}m_{y}=1,\;\mathrm{and}\;\sum_{y}d_{y}=0. \end{array}$$

By combining these constraints, we can obtain the inequality constraints on *d*:

(28) $$\begin{array}{c} \left\{\begin{array}{c} -m_{y}\leq d_{y}\leq m_{y},\;\mathrm{if}\;m_{y}<1/2\\ m_{y}-1\leq d_{y}\leq 1-m_{y},\;\mathrm{if}\;m_{y}\geq 1/2. \end{array}\right. \end{array}$$

According to Equation (26), we have the explicit forms of the mutual information and the recoverability for this case as follows:

$$\begin{array}{c} \left\{\begin{array}{c} I=p\sum_{y=1}^{3}\left(m_{y}+d_{y}\right)\log\frac{m_{y}+d_{y}}{m_{y}+\left(2p-1\right)d_{y}}+\left(1-p\right)\sum_{y=1}^{3}\left(m_{y}-d_{y}\right)\log\frac{m_{y}-d_{y}}{m_{y}+\left(2p-1\right)d_{y}},\\ R=2p\left(1-p\right)\sum_{y=1}^{3}d_{y}\log\frac{m_{y}+d_{y}}{m_{y}-d_{y}}, \end{array}\right. \end{array}$$

where the a priori probabilities $P_{X}\left(1\right)=p$ and $P_{X}\left(2\right)=1-p$. For no loss of generality, we set $m_{1}=m_{2}=m_{3}=1/3$ for the numerical calculations. Then, we can obtain the inequality constraints on *d* given in Equation (28) more explicitly:

(29) $$\begin{array}{c} -1/3\leq d_{1}\leq 1/3,-1/3\leq d_{2}\leq 1/3,\;\mathrm{and}\;-1/3\leq d_{1}+d_{2}\leq 1/3. \end{array}$$

**Figure 4:**
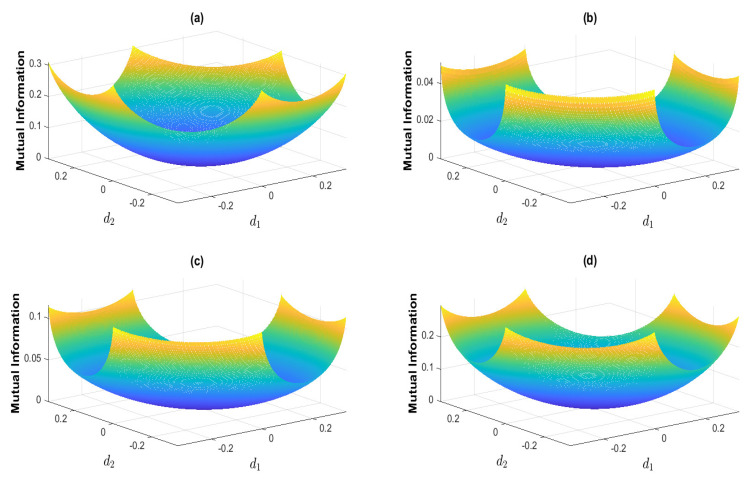
The mutual information as a function of the nonequilibrium strengths $\left(d_{1},d_{2}\right)$. The mutual information is calculated corresponding to the a priori probabilities given in Table 1.

**Figure 5:**
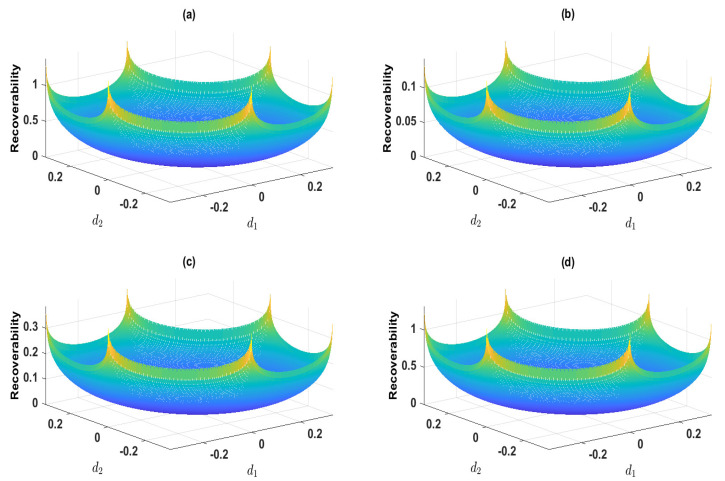
The recoverability as a function of the nonequilibrium strengths $\left(d_{1},d_{2}\right)$. Each recoverability is calculated corresponding to the a priori probability shown in Table 1.

**Figure 6:**
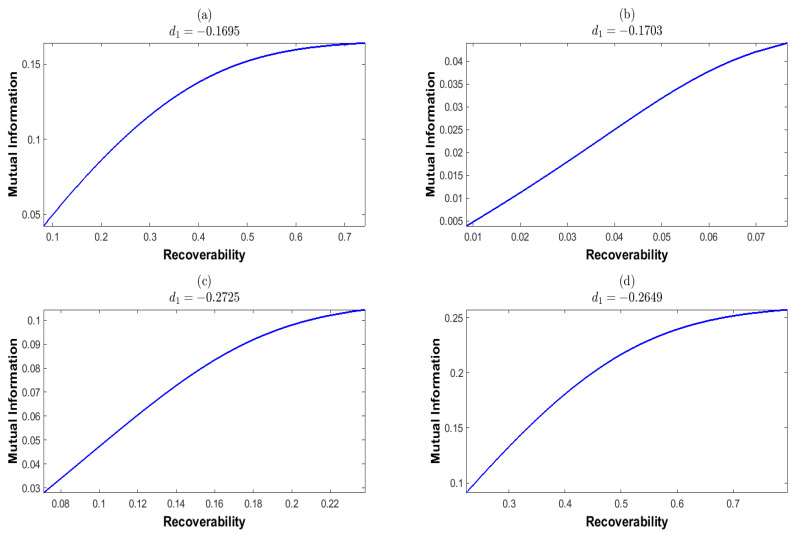
The mutual information as a function of the recoverability at fixed $d_{1}$. The mutual information and the recoverability are calculated corresponding to the a priori probabilities given in Table 1.

**Figure 7:**
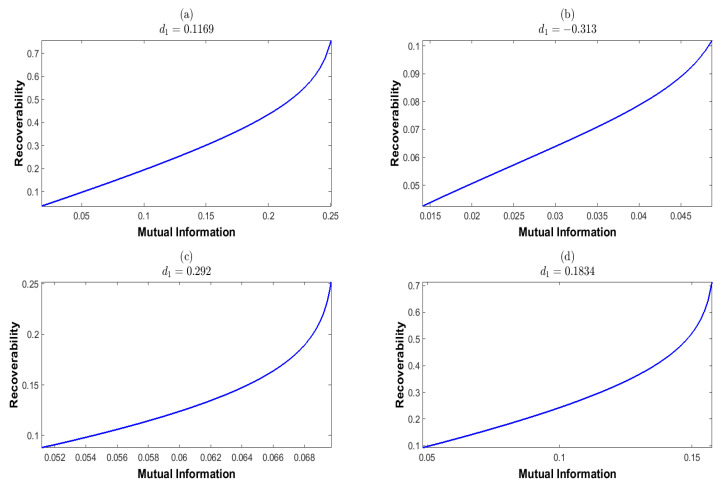
The recoverability as a function of the mutual information at fixed $d_{1}$. The mutual information and the recoverability are calculated corresponding to the a priori probabilities given in Table 1.

Here, we should note the identity $d_{3}=-d_{1}-d_{2}$, which is due to the equality constraint on *d* in Equation (27). This yields the last inequality for $d_{1}+d_{2}$. We then randomly select the a priori probability *p*, which is shown in Table 1. We plot the mutual information *I* and recoverability *R* as functions of the nonequilibrium strengths $\left(d_{1},d_{2}\right)$, which are shown in Figure 4 and Figure 5, respectively. These results show that *I* and *R* are both convex for $\left(d_{1},d_{2}\right)$. We next plot *I* as the function of *R* and *R* as the function of *I*, which are shown in Figure 6 and Figure 7, respectively. These results show that both the recoverability and the mutual information are monotonically increasing functions of each other. This indicates that the increasing information recoverability can enhance the information transfer rate, while the increasing information transfer rate enhances the information recoverability.

## 7. Conclusions

In this study, we investigated the nonequilibrium effects on the information recovery. By considering the information dynamics of the sequential information transfer (Equation (3)), we can quantify the recoverability of the source messages as the averaged log ratio of the transition probabilities of the channel (Equations (10) and (19)). We see that the difference between the transition probabilities works as the nonequilibrium strength behind the information dynamics (Equation (15)). The recoverability can be shown as the thermodynamic dissipation cost or the entropy production of the information dynamics (Equations (19) and (20)), driven by the nonequilibrium information driving force. This shows that the dissipation cost is essential for the information recoverability. The recoverability increases monotonically as the nonequilibrium information driving force or the nonequilibrium strength increases. On the other hand, as a function of the recoverability (Equation (19)), the mutual information also increases monotonically as the nonequilibrium strength increases. This demonstrates that the nonequilibrium cost can boost the information transfer from the thermodynamic perspective. In a similar spirit, increasing the information transfer rate can improve the information recoverability. The numerical results support our conclusions.

Finally, we discuss some examples that may have potential applications of the model and conclusion in the present work. As is well known, information transfer plays an important role in biology. For example, in biological sensory adaptation, which is an important regulatory function possessed by many living systems, organisms continuously monitor the time-varying environments while simultaneously adjusting themselves to maintain their sensitivity and fitness in response [36]. In this process, the information is transferred from the stochastic environments to the sensory neurons in the brain towards the objects that can be treated as the noisy channels. It is obvious that our model provides a simplified version of this process. The recoverability determines the accuracy of the response of the living system to the environment. A similar process also happens at the cellular level, where cells sense the information from the environment and transmit it towards signal transduction cascades to transcription factors in order to survive in a time-varying environment. As a response, a suitable gene expression is then initiated [37]. Similarly, in gene regulation, the time-varying transcription factor profiles are converted into distinct gene expression patterns through specific promoter activation and transcription dynamics [38,39,40,41]. In this situation, $X_{i-1}$ and $Y_{i}$ in Equation (3) can represent the input and the output states at a fixed time. In fact, because the transcription factor profiles are time-varying, the input signals and the corresponding output signals compose the complete trajectories of the input and output processes on a considered time interval [0,t]. In [42,43], it was shown that the mutual information can be used to quantify the cumulative amount of information exchanged along these trajectories. In the present work, we introduced the joint time sequence *Z* to describe the trajectories of the input and output processes. We believe that the model discussed in the present work can be applied to these above-mentioned situations. We will address the issues of the nonequilibrium recoverability in these examples in the near future.

## Figures and Tables

**Table 1:** The a priori probabilities $P_{X}\left(1\right)=p$ used for numerical illustrations.

	(a)	(b)	(c)	(d)
*p*	0.8212	0.0154	0.0430	0.1690

## Data Availability

Data available upon request.

## Appendix A

Here, we prove the convexity of the recoverability in Equation (10).

**Proof.** Select two different information driving forces $q_{Y|X}^{\left(1\right)}$ and $q_{Y|X}^{\left(2\right)}$. Then, the convex combination $q_{Y|X}^{\left(\lambda \right)}=\lambda q_{Y|X}^{\left(1\right)}+\left(1-\lambda \right)q_{Y|X}^{\left(2\right)}$ is also an information driving force, which is due to the nonnegativity and the normalization of the probability distributions. Then, we have

(A1) $$\begin{array}{ccc} \lambda R_{1}+\left(1-\lambda \right)R_{2} & = & \lambda \sum_{X_{i-1},X_{i-1}^{\prime },Y_{i}}P_{X}\left(X_{i-1}\right)P_{X}\left(X_{i-1}^{\prime }\right)q_{Y|X}^{\left(1\right)}\left(Y_{i}|X_{i-1}\right)\log\frac{q_{Y|X}^{\left(1\right)}\left(Y_{i}|X_{i-1}\right)}{q_{Y|X}^{\left(1\right)}\left(Y_{i}|X_{i-1}^{\prime }\right)}\\ & + & \left(1-\lambda \right)\sum_{X_{i-1},X_{i-1}^{\prime },Y_{i}}P_{X}\left(X_{i-1}\right)P_{X}\left(X_{i-1}^{\prime }\right)q_{Y|X}^{\left(2\right)}\left(Y_{i}|X_{i-1}\right)\log\frac{q_{Y|X}^{\left(2\right)}\left(Y_{i}|X_{i-1}\right)}{q_{Y|X}^{\left(2\right)}\left(Y_{i}|X_{i-1}^{\prime }\right)}\\ & \geq & \sum_{X_{i-1},X_{i-1}^{\prime },Y_{i}}P_{X}\left(X_{i-1}\right)P_{X}\left(X_{i-1}^{\prime }\right)q_{Y|X}^{\left(\lambda \right)}\left(Y_{i}|X_{i-1}\right)\log\frac{q_{Y|X}^{\left(\lambda \right)}\left(Y_{i}|X_{i-1}\right)}{q_{Y|X}^{\left(\lambda \right)}\left(Y_{i}|X_{i-1}^{\prime }\right)}\\ & = & R_{\lambda }. \end{array}$$

The log sum inequality $\sum_{i}a_{i}\log\frac{a_{i}}{b_{i}}\geq \left(\sum_{i}a_{i}\right)\log\frac{\sum_{i}a_{i}}{\sum_{i}b_{i}}$ was applied in Equation (A1) [31]. Thus, *R* is convex with respect to $q_{Y|X}$ with the unique minimum point R=0 if and only if $q_{Y|X}\left(Y_{i}|X_{i-1}\right)=q_{Y|X}\left(Y_{i}|X_{i-1}^{\prime }\right)$ for all $Y_{i}$ and $X_{i-1}^{\prime }\neq X_{i-1}$. Note that, in this paper, we chose the conventional rule that the convex function is a synonym for the concave-up function with a positive second derivative. This completes the proof. Since both the mutual information *I* and the recoverability *R* are convex functions of the transition probabilities $q_{Y|X}$, then both of them are the convex functions of the affine transformation of $q_{Y|X}$, i.e.,

$$\begin{array}{c} \left\{\begin{array}{c} d_{Y_{i}}\left(X_{i-1},X_{i-1}^{\prime }\right)=\frac{1}{2}\left[q_{Y|X}\left(Y_{i}|X_{i-1}\right)-q_{Y|X}\left(Y_{i}{|}^{\prime }X_{i-1}\right)\right],\\ m_{Y_{i}}\left(X_{i-1},X_{i-1}^{\prime }\right)=\frac{1}{2}\left[q_{Y|X}\left(Y_{i}|X_{i-1}\right)+q_{Y|X}\left(Y_{i}{|}^{\prime }X_{i-1}\right)\right]. \end{array}\right. \end{array}$$

This means that both *I* and *R* are convex functions of the difference *d* by fixing every *m*. □
